# Complete Genome Sequence of the Piezophilic, Mesophilic, Sulfate-Reducing Bacterium *Desulfovibrio hydrothermalis* AM13^T^

**DOI:** 10.1128/genomeA.00226-12

**Published:** 2013-02-21

**Authors:** Boyang Ji, Gregory Gimenez, Valérie Barbe, Benoît Vacherie, Zoé Rouy, Amira Amrani, Marie-Laure Fardeau, Philippe Bertin, Didier Alazard, Sabine Leroy, Emmanuel Talla, Bernard Ollivier, Alain Dolla, Nathalie Pradel

**Affiliations:** a Aix-Marseille Université, CNRS, Laboratoire de Chimie Bactérienne (LCB), UMR 7283, Marseille, France; b UMR 6236, Faculté de Médecine, Marseille, France; c CEA, Institut de Génomique/Genoscope, Laboratoire de Finition, Évry, France; d Aix-Marseille Université, Université du Sud Toulon-Var, CNRS, Institut National des Sciences de l’Univers (INSU), Institut de Recherche pour le Développement (IRD), Mediterranean Institute of Oceanography (MIO), UM110, Marseille, France; e UMR 7156, CNRS, Université Louis Pasteur, Strasbourg, France; f INRA, UR454 Microbiologie, Saint-Genès Champanelle, France

## Abstract

*Desulfovibrio hydrothermalis* AM13^T^ is a piezophilic, mesophilic, hydrogenotrophic sulfate-reducing bacterium collected from a deep-sea hydrothermal chimney on the East Pacific Rise (2,600 m depth, 13°N). We report the genome sequence of this bacterium, which includes a 3,702,934-bp chromosome and a circular plasmid of 5,328 bp.

## GENOME ANNOUNCEMENT

Deep-sea sulfate-reducing microorganisms must be considered as key actors in Earth’s sulfur cycles. Metagenomic studies have revealed that sulfate-reducing bacteria (SRB) are largely represented among the microorganisms that inhabit deep-sea environments (1–3). However, only a few SRB have been isolated from deep-sea environments (4–9). Among them, the *Desulfovibrio* genus is well-represented, with *Desulfovibrio profundus*, *Desulfovibrio piezophilus*, and *Desulfovibrio hydrothermalis* having been isolated previously (7–9). Here, we report the genome sequence of *D. hydrothermalis*, a piezo-mesophilic, hydrogenotrophic SRB isolated from a deep-sea hydrothermal chimney sample collected at a depth of 2,600 m (13°N on the East Pacific Rise) (7). It represents, with *D. piezophilus* (10), the second known genome of piezophilic bacteria in the genus *Desulfovibrio*, and thus gives the opportunity to specify the systems that are involved in the piezophilic lifestyles of *Desulfovibrio* species.

Genome sequencing was performed by the Genoscope (CEA, Évry, France), using 454-sequencing method (Roche) and Solexa technology. A mate-paired genomic library with 8-kb insert size was prepared according to the manufacturer’s protocols (454 Life Sciences Corporation, Branford, CT). Approximately 19-fold coverage of GS FLX Titanium (http://www.roche.com/) reads was assembled using Newbler. Gaps between contigs were closed with 1,038 PCR amplifications and the Consed editing software (http://www.phrap.org/). For the quality assessment, approximately 1,000-fold coverage of paired-end Illumina reads (36 bp) was mapped onto the whole-genome sequence.

The genome consists of two contigs of 284,269 bp and 3,418,665 bp, for a total of 3,702,934 bp, and of a circular plasmid of 5,328 bp. The average G+C contents for the chromosome and the plasmid DNA are 45.1% and 51.1%, respectively. A total of 3,543 coding DNA sequences (CDSs) were predicted, as well as 7 pseudogenes, 6 miscellaneous RNAs (misc-RNA), 5 disrupted rRNA operons, and 77 tRNA genes.

Phylogenetic analysis based on 16S rRNA shows that the closest sequenced genomes to *D. hydrothermalis* are those of *Desulfovibrio salexigens* and *Desulfovibrio aespoensis*. Reciprocal best BLAST analysis indicated that *D. hydrothermalis* shares 2,824 and 2,069 orthologous proteins with *D. salexigens* and *D. aespoensis*, respectively, and 1,974 proteins are common to the three species.

Like other *Desulfovibrio* species, *D. hydrothermalis* possesses all the genes necessary for the sulfate-reduction pathway. Fatty acid membrane composition has been shown to play an important role in hydrostatic pressure adaptation (11). In. *D. hydrothermalis*, all fatty acid biosynthesis pathways are present. Interestingly, three copies of the *fabF* gene (DESAMv2_20244, DESAMv2_21138, and DESAMv2_21153), encoding a β-ketoacyl-acyl carrier protein (ACP) synthase II involved in monounsaturated fatty acid production and suggested to be involved in hydrostatic pressure adaptation (11), are found in the genome. Thus, the three copies of this gene might play a pivotal role in the adaptation strategy of *D. hydrothermalis* to hydrostatic pressure.

Comparative genomics, including the use of other *Desulfovibrio* species, will give insights into the adaptation of *Desulfovibrio* species to the piezophilic lifestyle that they encounter in the deep-sea biosphere.

### Nucleotide sequence accession numbers. 

The final annotated genome of *D. hydrothermalis* is available in EMBL under the accession no. FO203522 (for the chromosome) and FO203523 (for the plasmid).
